# Supplementary material for: Pathogens of Medical Importance Identified in Hospital-Collected Cockroaches: A Systematic Review
Source: Microorganisms. 2025 Feb 4;13(2):337. doi: 10.3390/microorganisms13020337 (PMC11858024; doi:10.3390/microorganisms13020337)
Supplement: Supplementary file 1 [file microorganisms-13-00337-s001.zip › microorganisms-3348464-supplementary.pdf]

Table S1. Studies analyzed in the systematic review, 2000-2024

Author, year, reference number	Country/ Developed Category	Microorganisms isolated and identified from cockroaches	Methods used in the identification of microorganisms [IM], determination of antimicrobial susceptibility profile [PSA]	Antibiotic Resistance Mechanisms [ARM].
Prado et al., 2002 (12)	Brazil/Developing Country	**Bacteria:** *E. coli, K. pneumoniae, E. cloacae, S. marcescens, H. alvei, E. gegorviae, Serratia* spp., *K. oxytoca, P. vulgaris, Morganella morganii*	**IM (Bacteria):** Seeding in culture media (general and differential), Gram stain (TG) and conventional biochemical tests (CBT). **PSA (Bacteria):** Disc Diffusion Method (Kirby Bauer).	No detected
Gliniewicz et al. , 2003 (13)	Poland/Developing Country	**Bacteria:** *S. salivarius, S. oestibularis, K. oxytoca, Enterococcus avium, Enterococcus salivarium, S. hominis, E. cloacae, S. equorum, S. epidermis, Micrococcus luteus, C. freundii, S. marcescens, P. aeruginosa*	**IM (Bacteria):** Seeding in culture media (general and differential), Gram stain (TG) and conventional biochemical tests (CBT). **PSA (Bacteria):** Disc Diffusion Method (Kirby Bauer).	Inactivation of beta-lactam drugs by the expression of extended-spectrum beta-lactamases (ESBL).
Fathpour et al.,2003 (11)	Iran /Developing Country	**Bacteria:** *Salmonella* spp.	**IM (Bacteria):** Seeding in culture media (general and differential), Gram stain (TG) and conventional biochemical tests (CBT). **PSA (Bacteria):** Disc Diffusion Method (Kirby Bauer).	No detected
Pai et al., 2004 (22)	Taiwan/Develop Country	**Bacteria:** *Streptococcus*. spp., *Bacillus* spp., *Bacillus subtilis, S. aureus, S. epidermidis, K. oxytoca, K. pneumoniae, E. coli, E. agglomerans, E. odorifera, E. aerogenes, E. cloacae, E. gergoviae, E. dissolvens, S. marcescens, S. odorifera, S. rubidaea, Pasteurella canis, C. freundii, C. diversus, P. mirabilis, P. vulgaris, H. alvei, P. aeruginosa, P. mallei, P. cepacia* **Fungi:** *C. glabrata, C. guilliermondii, C. krusei, C. lambica, C. lusitaniae, C. parapsilosis*, C. *pintolopesii, C. rugosa, C. tropicalis, C. sake, Hansenula anomala, A. flavus*	**IM (Bacteria**): Seeding in culture media (general and differential), Gram stain (TG) and conventional biochemical tests (CBT). PSA (Bacteria): Disc Diffusion Method (Kirby Bauer). **IM (Fungi)**: Seeding in Sabouraud agar mediulm and observation of macro and microscopic morphological characteristics of the sample.	No detected

Tachbele et al .,2004<br>(23)	Ethiopia/ Developing Country	**Bacteria:** *Salmonella* spp, *Shigella* spp, *S. aureus*, *E. coli* 0157:H7, *Bacillus cereus*	**IM (Bacteria):** Seeding in culture media, biochemical and serological tests,<br>**PSA (Bacteria):** Disc Diffusion Method (Kirby Bauer).	No detected
Prado et al., 2006<br>(24)	Brazil/ Developing Country	**Bacteria:** *S. coagulase-negative*, *E. aerogenes*, *S. marcescens*, *H. alvei*, *E. cloacae*, *E. gergoviae*, *Serratia* spp.<br>**Fungi:** Yeast and Filamentous fungi.	**IM (Bacteria):** Seeding in culture media (general and differential) and PBC.<br>**PSA (Bacteria):** Disc Diffusion Method (Kirby Bauer).<br>**IM (Fungi):** Seeding in Sabouraud agar medium and observation of macro and microscopic morphological characteristics of the sample.	No detected
Elgderi et al., 2006<br>(14)	Libya/ Developing Country	**Bacteria:** *E. coli*, *K. pneumoniae*, *K. oxytoca*, K. *ornithinolytica*, *E. cloacae*, *E. aerogenes*, *Pantoea* sp, *C. freundii*, *C. braakii*, *C. youngae*, C. *amalonaticu*, *S. marcescens*, *S. liquefaciens*, *P. mirabilis*, *P. vulgaris*, *M. morganii*, *H. alvei*, *Buttiauxlla agrestis*, *Aeromonas hydrophila*, *Aeromonas caviae*, *P. aeruginosa*, *Acinetobacter* sp.,<br>*Streptococcus* sp.	**IM (Bacteria):** Seeding in culture media (general and differential), PBC y API.<br>**PSA (Bacteria):** Disc Diffusion Method (Kirby Bauer).	No detected
Tachbele et al., 2006<br>(25)	Ethiopia/ Developing Country	**Bacteria:** *Shigella flexneri*, *E. coli* O15717, *S. aureus*, *Bacillus cereus*.	**IM (Bacteria):** Seeding in culture media (general and differential), TG, PBC and Serological Tests.<br>**PSA (Bacteria):** Disc Diffusion Method (Kirby Bauer).	No detected
Pancer et al. , 2006<br>(26)	Poland /Developing Country	**Bacteria:** *E. cloacae*	**IM (Bacteria):** the method used is not shown.<br>**PSA:** diffusion test on Muller-Hinton Agar (Disc Diffusion Method).	Inactivation of beta-lactam drugs by the expression of extended-spectrum beta-lactamases (ESBL).

Salehzadeh et al., 2007 (27)	Iran/ Developing Country	**Bacterias:** *E. coli*, *Haemophilus* spp., *S. hemolítico* group A y B. **Fungi:** *Candida* spp., *Mucor* spp., *Aspergillus niger*, *Rhizopus* spp., *Penicillium* spp., *Aspergillus fumigans* **Helminths:** *Enterobius vermicularis*, *Ascaris* spp.	**IM (Bacteria):** Seeding in culture media (general and differential), TG y PBC. **PSA (Bacteria):** the method used is not shown.	No detected
Miranda et al., 2008 (28)	Brazil/ Developing Country	**Bacteria:** *Salmonella* spp., *E. coli*, *C. freundii*, *H. alvei*, *S. aureus*, *E. aerogenes*, *Serratia* spp.	**IM (Bacteria):** Seeding in culture media (general and differential), TG y PBC. **PSA (Bacteria):** the method used is not shown.	No detected
Saitou et al., 2009 (18)	Japan/ Developed Country	**Bacteria:** *P. aeruginosa*	**IM (Bacteria):** Seeding in culture media (general and differential), PBC y API. **PSA (Bacteria):** Using the Etest.	No detected
Tilahun et al., 2012 (15)	Ethiopia/ Developing Country	**Bacteria:** *K. oxytoca*, *K. pneumoniae*, *E. cloacae*, *C. diversus*, *P. aeruginosa*, *Providencia rettgeri*, *K. ozaenae*, *E. aerogenes*, *S. aureus*, *E. coli*, *Shigella flexneri*, *Enterococcus faecalis*.	**IM (Bacteria):** Seeding in culture media (general and differential), TG y PBC. **PSA (Bacteria):** Disc Diffusion Method (Kirby Bauer).	No detected
Tetteh-Quarcoo et al., 2013 (29)	Ghana /Developing Country	**Bacteria:** *K. pneumoniae*, *E. coli*, *P. vulgaris*, *C. ferundii*, *E. cloacae*, *P. aeruginosa*, *Enterococcus faecalis*, *K. oxytoca*. **Helminths:** *Ancylostoma duodenale*, *Taenia* spp. **Virus:** Rotavirus	**IM (Bacteria):** Seeding in culture media (general and differential), TG y PBC. **IM (Helminths):** Staining of the sample with lugol and observation under a light microscope. **IM (Rotavirus):** ELISA **PSA (Bacterias):** Disc Diffusion Method (Kirby Bauer).	No detected
Motevalli-Haghi et al., 2014 (30)	Iran/ Developing Country	**Fungi:** *Candida* spp., *Rhodotorula* spp., *Aspergillus* spp., *Fusarium* spp., *Penicillium* spp. *Geotrichum* spp., *Alternaria* spp., *Cladosporium* spp., *Trichoderma* spp., *Mucor* spp., *Chrysosporium* spp.	**IM (Fungi):** Seeding in Sabouraud's dextrose agar with chloramphenicol, tube germination test (yeast) and observation of macro and microscopic morphological characteristics of the sample.	No detected

Reza et al., 2014 (31)	Iran/ Developing Country	**Bacteria:** *Pseudomonas* , *Enterobacter*, *Bacillus*, *Klebsiella*, *Citrobacter*, *Proteus*, *Acinetobacter* , *Providentia*, *E. coli*, *Moraxella*, *S. saprophiticus*	**IM (Bacteria):** Standard Culture Methods. **PSA (Bacterias):** Disc Diffusion Method (Kirby Bauer).	No detected
Suresh et al.; 2015 (32)	India/ Developing Country	**Bacteria:** *Salmonella* B, *Salmonella* D, *Salmonella* E, *Shigella* B, *E. coli*, *S. aureus*.	**IM (Bacteria):** Seeding in culture media (general and differential), TG, PBC y Pruebas Serológicas. **PSA (Bacteria):** Disk Diffusion Method (Kirby Bauer).	No detected
Cazorla et al.; 2015 (33)	Venezuela/ Developing Country	**Protozoos:** *Entamoeba blattae*, *Nycthoterus ovalis*, *Leptomonas* spp., *Cyclospora* spp., *Entamoeba coli*, *Cystoisospora* spp., *Lophomonas blattarum*, *Lophomonas striata* **Helminths:** *Enterobius vermicularis*, *Thelastoma* spp., *Hammerschmidtiella* spp.	**IM (Protozoans and Helminths**): Direct parasitologic methods: samples paired in saline, Lugol stained, then light microscoped.	No detected
Jabber et al ., 2015 (34)	Iraq/ Developing Country	**Bacteria:** *A.hydrophila/coviae/sobria*, *C. freundii*, *C. brakii*, *C .intermedies*, *E. coli*, *E. aerogenes*, *E.cloacae*, *E. sakazakii*, *K .oxytoca*, *K. neumoniae*, *P.mirabilis*, *P. vulgaris*, *P. rettegri*, *P. aerugenosa*, *Rao. ornithinolyticae*, *Sal*. spp , *S. marcescens*, *S. liquificiaus*, *S. odorifera*, *Shi*. spp	**IM (Bacteria):** Seeding in culture media (general and differential) **PSA:** Dilution Method on Mueller -Hinton Agar	No detected
Loucif et al., 2016 (16)	Argelia/ Developing Country	**Bacteria:** *C. amalonaticus*, *C. farmeri*, *C. freundii*, *C. koseri*, *E. cloacae*, *E. kobe*i, *K. oxytoca*.	**IM (Bacteria):** API 20E system (bioMérieux, France) and matrix-assisted laser desorption ionization–time of flight mass spectrometry. **PSA (Bacteria):** Disc Diffusion Method (Kirby Bauer).	Inactivation of beta-lactam drugs by the expression of extended-spectrum beta-lactamases (ESBL).

Ikechukwu et al., 2017 (35)	Nigeria/ Developing Country	**Bacteria:** E. coli, Salmonella spp., Shigella spp.	**IM (Bacteria):** Seeding in culture media (general and differential), TG y PBC. **PSA (Bacteria):** Disc Diffusion Method (Kirby Bauer).	Inactivation of beta-lactam drugs by the expression of extended-spectrum beta-lactamases (ESBL) Mechanism of Target site alteration that protects the ribosome.
Abdolmaleki et al., 2017 (19)	Iran/ Developing Country	**Bacteria:** *S. aureus*	**IM (Bacterias):** Seeding in culture media (general and differential), TG y PBC. **PSA (Bacterias):** Disc Diffusion Method (Kirby Bauer) and PCR.	Inactivation of beta-lactam drugs by the expression of extended-spectrum beta-lactamases (ESBL), Mechanism of Target site alteration that protects the ribosome.
Loucif et al., 2017 (36)	Argelia/ Developing Country	**Bacteria:** *P. putida*	**IM (Bacteria):** Vitek 2 **PSA (Bacterias):** Disc Diffusion Method (Kirby Bauer), PCR and Vitek.	Inactivation of beta-lactam drugs by the expression of extended-spectrum beta-lactamases (ESBL).
Martínez-Girón et al.,2017 (37)	Spain/ Developed Country	**Protozoos:** *Lophomonas blattarum*, *Nyctotherus* sp, *Gregarina* sp, *Amoeba* sp.	**IM (Protozoans):** Giemsa Stain and the Papanicolaou Method.	No detected
Al-Marjani et al ., 2017 (38)	Iraq/ Developing Country	**Bacteria:** *K.pneumoniae*, Coagulase, Staphylococci, *P. aeruginosa*, *E. coli*	**IM (Bacteria):** Seeding in culture media (general and differential). **PSA (Bacterias):** Disc Diffusion Method (Kirby Bauer)	Inactivation of beta-lactam drugs by the expression of extended-spectrum beta-lactamases (ESBL).

Naher et al.,2018 (39)	Bangladesh/ Developing Country	**Bacteria:** *Salmonella* spp, *Shigella* spp, *P. aeruginosa*, *Klebsiella* spp, *Proteous*. spp, *S .aureus*, *S. epidermidis*, *Bacillus cereus*, *Enterobacter* spp, *E. coli*, *S. pyogenes*, Haemophilus spp. **Fungi and Parasites**	**IM (Bacteria):** colonial morphology, Gram Staining and a Battery of Biochemical Test, BBL Antisera. **PSA (Bacterias):** Disc Diffusion Method (Kirby Bauer) **IM (Fungi):** Seeding in Sabouraud's dextrose agar with chloramphenicol, tube germination test (yeast) and observation of macro and microscopic morphological characteristics of the sample. **IM (Protozoans and Helminths**): Direct parasitologic methods: samples paired in saline, Lugol stained, then light microscoped to observed characteristic's morphological.	No detected
Astiti et al., 2018 (40)	Indonesia/ Developing Country	**Bacteria:** *E. coli*, *K. ozaenae*, *Salmonella arizonae*, *Salmonella choleraesuis*, *Salmonella* sp, *Salmonella simultaneous*	**IM (Bacteria):** Seeding in culture media (general and differential). **PSA (Bacterias):** Disc Diffusion Method (Kirby Bauer)	No detected
Abdolmaleki et al.,2019 (6)	Iran/ Developing Country	**Bacteria:** *S. aureus*	**IM(Bacteria):** Methicillin-resistant S.aureus bacteria were confirmed using cefoxitin (30 g) andoxacillin (1 g) sus ceptibility tests and PCR. **PSA (Bacteria):** PCR	Inactivation of beta-lactam drugs by the expression of extended-spectrum beta-lactamases (ESBL) Mechanism of Target Site alteration that protects the ribosome.
Nazari et al., 2020 (41)	Iran/ Developing Country	**Bacteria:** *E. coli*, S. coagulasa-negativa, *Proteous* spp., *Enterococcus* spp., *Micrococcus* spp., *Pseudomona* spp., *Serratia* spp., *Streptococcus*. B, *Streptococcus*. A, *S. aureus*.	**IM (Bacteria):** Seeding in culture media (general and differential), TG y PBC. **PSA (Bacteria):** Disc Diffusion Method (Kirby Bauer).	No detected

Khodabandeh et al., 2020 (53)	Iran/ Developing Country	**Fungi:** *Aspergillus niger*, *Rhizopus* spp., *Penicillium* spp., *Mucor* spp., *Candida glabrata*, *Candida krusei*	**IM (Protozoans):** Seeding in different culture media; observation of macro and microscopic morphological characteristics of the sample.	No detected
Chehelgerdi et al., 2021 (20)	Iran/ Developing Country	**Bacteria:** *S. pneumoniae*, *S. pyogenes*, *S. agalactiae*	**IM (Bacterias):** Seeding in culture media (general and differential), TG, PBC y PCR. **PSA (Bacteria):** Disc Diffusion Method (Kirby Bauer) and PCR	Inactivation of beta-lactam drugs by the expression of extended-spectrum beta-lactamases (ESBL). Target Site Modification.
Montevalli-Haghi et al., 2021 (43)	Iran/ Developing Country	**Protozoos:** *Gregarina* sp., *Lophomonas blattarum*, *Entamoeba* sp, *Blastocystis* sp, *Nyctotherus* sp.	**IM (Protozoans):** Direct parasitologic methods: samples paired in saline, Lugol stained, then light microscoped.	No detected
Adegoke et al., 2021 (44)	Nigeria/ Developing Country	**Bacteria:** *S. aureus*, *S. intermedius*	**IM (Bacterias):** standard microbiological methods. **PSA:** Kirby-Bauer disc diffusion technique	Inactivation of beta-lactam drugs by the expression of extended-spectrum beta-lactamases (ESBL).
Fotouhi-Ardakani et al., 2021 (45)	Iran/ Developing Country	**Virus:** SARS-CoV-2(Coronavirus)	**IM (virus):** Multiplex one-step qPCR Technique	No detected
Landolsi et al., 2022 (46)	Tunisia/ Developing Country	**Bacteria:** *K. pneumoniae*, *E. coli*, *E. cloacae*, *C. sedlaki*	**IM (Bacteria):** Seeding in different culture media and mass spectrometry (MALDI-TOF). **PSA (Bacteria):** Disc Diffusion Method (Kirby Bauer) and PCR.	Inactivation of beta-lactam drugs by the expression of extended-spectrum beta-lactamases (ESBL) Target Site Modification mediated by the mcr-1 gene
Seyfi at el., 2022 (47)	Iran / Developing Country	**Bacteria:** *E. coli*	**IM (Bacteria):** Seeding in culture media (general and differential).	Inactivation of beta-lactam drugs by the expression of

			**PSA:** DisK Diffusion Method (Kirby Bauer).	Betalactamases and extended-spectrum Beta-Lactamases (ESBL)
Turki et al.,2023 (48)	Iraq/ Developing Country	**Bacteria:** *E. coli*, *Bacillus* spp, *Klebsiella* spp , *Enterococcus* spp, *Acinetobacte*r species , Coagulase-negative Staphylococci, *Proteous*. spp, *E. aerogenes*, *P. aeruginosa*	**IM (Bacteria):** Seeding in different culture Medium. To isolate gram-negative bacteria, tests such as gram staining, fermentation of sugars, and motility tests such as TSI, SIM, Simon citrate, were done. Noteworthy, different tests such as gram staining, oxidase, and catalase tests, sensitivity to antibiotics, fermentation of sugars (mannitol) to isolate Gram-positive bacteria<br>**PSA (Bacteria):** DisK Diffusion Method (Kirby Bauer).	No detected
Kalantari et al., 2023 (49)	Iran/ Developing Country	**Virus:** SARS-CoV-2(Coronavirus)	**IM (virus):** RT-qPCR.	No detected
Madani et al., (2023) (50)	Iran/ Developing Country	**Fungi:** *Aspergillus niger*, *Penicillium italicum*, *Mucor plumbeus*, *Rhizopus oryzae*, *Pichia kudriavzevii*, *Candida glabrata*, *Pichia kluyveri*, *Candida viswanathi*	**IM (Fungi):** Seeding in general and different culture Medium. Observed their macroscopic and microscopic characteristics and used PCR.	No detected
Davari et al., 2023 (51)	Iran/ Developing Country	**Bacteria:** *E. coli*, *Bacillus* spp, *Klebsiella* spp , *Enterococcus* spp, *Acinetobacter* spp., *Coagulase negative Staphylococci*, *Proteous*. spp., *E. aerogenes*, *P. aeruginosa*	**IM (Bacteria):** Seeding in culture media (general and differential) and use of Battery Biochemical Test.<br>**PSA(Bacteria):** Disc Diffusion Method (Kirby Bauer).	No detected
Veysi et al., 2023 (52)	Iran/ Developing Country	**Bacteria:** *E. coli*, *P. aeruginosa*, *Salmonella* serogroup D, *Salmonella* serogroup A, *Shigella sonnei*, *Shigella dysenteriae*, *P. vulgaris*, *P. mirabilis*, *K. pneumoniae*, *K .oxytoca*,	**IM (Bacteria):** Seeding in culture media (general and differential) and use of Battery Biochemical Test.	No detected

		D. *agglomerans*, *S. aureus*, *Coccus* Positive, *C. freundii*, *C. disversus*, *H. alvei*	**PSA (Bacteria):** Disc Diffusion Method (Kirby Bauer)	
Cevahir et al ., 2024 (53)	Turkey/ Developing Country	**Protozoos:** *Blastocystis* sp, *Eimeria* spp, *Crystosporidium* spp. **Helminth:** *Toxocara* spp, *Ascaris lumbricoide*	**IM(Protozoos and Helminth):** Direct parasitologic methods: samples paired in saline, Lugol stained, then light microscoped to observed characteristic's morphological	No detected
Saberi et al., 2025 (54)	Iran/ Developing Country	**Bacteria:** *S. aureus*	**IM (Bacteria):** Seeding in culture media (general and differential) **PSA (Bacteria):** Disc Diffusion Method (Kirby Bauer)	Biofilm Production
